# The effects of perceived chronic stress and work-related factors on the risk of incident depression among German general practice personnel: a causal pathway approach using IMPROVE*job* study data

**DOI:** 10.1186/s12889-026-26366-9

**Published:** 2026-01-31

**Authors:** Arezoo Bozorgmehr, Mona Ahmed, Julian Göbel, Benjamin Aretz, Birgitta Weltermann

**Affiliations:** 1 Institute of General Practice and Family Practice, University Hospital Bonn, University of Bonn, Venusberg-Campus 1, Bonn, 53127 Germany; 2 Institute for Digitalization and General Medicine, Center for Rare Diseases Aachen (ZSEA), Medical Faculty, RWTH Aachen University, Pauwelsstraße 30, Aachen, 52074 Fed. Rep. Germany; 3 Institute for Medical Biometry, Informatics and Epidemiology (IMBIE), University Hospital Bonn, University of Bonn, Bonn, Fed. Rep. Germany

**Keywords:** Family physician, Primary care physician, Practice assistant, Coping, Work commitment, Work-related behavior, General practices, Leadership, Workload, Workplace, Surveys and questionnaire, Germany

## Abstract

**Background:**

The increasing workload faced by general practitioners (GPs) and practice assistants (PrAs) contributes to chronic stress and heightens the risk of depression. Various work-related factors, including work-privacy conflict (WPC), may further exacerbate this risk. This study explores the role of work-related factors, focusing on WPC, as mediators in the association between perceived chronic stress and the risk of incident depression among German GP practice personnel.

**Methods:**

This study included 366 German GPs and PrAs using baseline and follow-up data from the IMPROVE*job* trial. We assessed the risk of incident depression (Cut-off = 50) through changes in the World Health Organization Well-being Index (WHO-5) and measured perceived chronic stress using the Trier Inventory for the Assessment of Chronic Stress Screening Scale (TICS-SSCS). Work-related factors were evaluated, with a specific focus on WPC, using the corresponding scale from the Copenhagen Psychosocial Questionnaire (COPSOQ III). A causal mediation analysis was performed to examine how work-related factors mediate the effect of perceived chronic stress on depression risk.

**Results:**

Increased perceived chronic stress was observed in 43.64% of participants, while 56.36% reported stable or decreased stress levels from baseline to follow-up. This increase in perceived chronic stress was associated with heightened reported work-privacy conflict (β = 8.99, *p* = 0.01), though higher baseline levels of work-privacy conflict appeared to mitigate this effect (β = -0.35, *p* < 0.001). Reported Work-privacy conflict increased for 43.22% of participants, remained stable for 28.39%, and decreased for another 28.39%. Moreover, heightened reported work-privacy conflict correlated with a greater risk of incident depression (logit β = 0.20, *p* = 0.015; OR = 1.02 per point, 1.22 per 10-point increase), with baseline conflict levels showing smaller subsequent increases (β = -0.35, *p* < 0.001), likely reflecting regression to the mean/ceiling effect. The risk of incident depression was identified in 19.49% of participants, while 80.51% did not develop depressive symptoms. Causal mediation analysis further revealed that work-privacy conflict partially mediate the relationship between chronic stress and the risk of incident depression, with both a direct effect ($\:\zeta\:$ = 0.17, *p* = 0.002) and a mediation effect ($\:{{\updelta\:}}_{i}$ = 0.03, *p* = 0.020) contributing to this association. Additionally, no significant associations were found between personal or work-related factors (e.g., age, gender, work time) and incident depression.

**Conclusion:**

This study emphasizes the need to address work-privacy conflict as a key factor in reducing the mental health burden associated by chronic stress among general practice personnel.

## Introduction

### Relevance of the topic

The increasing workload among general practice personnel presents a significant challenge for the German healthcare system. General practitioners (GPs) and practice assistants (PrAs) face high daily patient volumes and additional pressures due to recent legislative changes, increased administrative and documentation requirements, and the complexities of practice management [1, 2]. These demands are further compounded by evolving patient expectations and behaviors regarding health and healthcare utilization [2]. Together, these factors contribute to a highly complex and demanding work environment for GP practice personnel. The aging of the healthcare workforce enhances this challenge, with a significant proportion of professionals aged 50 and older [3]. The shortage of skilled professionals in medical practices presents a precarious situation [4].

These developments are significant for the German healthcare system since general practices play a crucial role in providing primary care for patients seeking medical assistance, managing diverse responsibilities that encompass both patient care and administrative duties [5].

Chronic stress in general practice personnel stems, amongst others, from the relentless pressures of patient care, administrative responsibilities, and a rapidly evolving healthcare landscape. Long working hours, heavy workloads, and frequent exposure to emotionally challenging situations take a toll on their well-being. Chronic stress may not only elevate the risk of burnout, depression, and anxiety but also reduce job satisfaction and impairs job performance. Thielemann et al. demonstrated a positive selection bias, showing that many general practitioners developed effective coping mechanisms and resilience. This underscores their capacity to counteract the negative effects of chronic stress, balancing the predominantly adverse outcomes often highlighted [6].

This deterioration in mental health affects both the quality of life for general practice personnel and the standard of care they can provide to patients [7–10].

The risk of incident depression is a significant mental health concern, not only among the general population but also for general practice personnel [11–13]. The emotional demands of patient care, combined with broader professional stress, can increase this risk. Gaining insight into the factors and pathways contributing to the onset of depression among GPs and PrAs is essential for developing targeted interventions to alleviate their mental health burden.

In modern work environments, stress and work-related factors like perceived work-privacy conflict (WPC) are major challenges. Stress affects well-being through physiological, psychological, and behavioral responses, while the work-privacy conflict arise, among others, from blurred boundaries between work and personal life, often worsened by constant connectivity [7, 9, 11, 14–16]. Understanding these mechanisms is key to addressing the impact of chronic stress and fostering a healthier work-life balance for general practice personnel.

### The IMPROVE*job* trial

The IMPROVE*job* trial was designed to improve psychosocial work conditions for German general practice personnel. The main objectives of this cluster-randomized controlled trial (cRCT) were to enhance job satisfaction and to reduce perceived chronic stress among the target group. The intervention included live training sessions, an online toolbox, and trained facilitators, and was conducted by clinician-scientist experts from family medicine, as well as specialists in occupational medicine, psychosomatic medicine, operations research, epidemiology, and health promotion. The study protocol and baseline data have been published, showing that general practice personnel reported both high job satisfaction and high levels of perceived chronic stress [17, 18].

### Theory and hypothesis

Based on the aforementioned information, we hypothesize a correlation between perceived chronic stress, the risk of incident depression, and various work-related factors, with work-privacy conflict expected to play a mediating role. We propose that perceived work-privacy conflict may contribute to the development of depression when perceived chronic stress is present [16]. This study aims to elucidate the mechanisms linking perceived chronic stress and depression within this occupational group, thereby contributing to a more comprehensive understanding of mental health risks in healthcare settings. The findings may inform targeted interventions to enhance work sustainability, ultimately supporting both personnel well-being and the quality of patient care in general practice. While this study focuses on WPC as a mediator on the pathway from perceived chronic stress to incident depression, WPC itself is also conceptualized as a relevant source and amplifier of chronic stress in contemporary work environments. The analytic framework assumed a causal pathway from increased perceived chronic stress via WPC to incident depression, but this relationship is likely bidirectional in practice. WPC may not only mediate but also contribute to the emergence and maintenance of chronic stress, which should be examined in future studies using designs that allow for reciprocal effects [19].

## Materials and methods

### Study design and ethical considerations

This study used the baseline and follow-up data of the IMPROVE*job* trial conducted in the federal state of North Rhine-Westphalia, Germany, with personnel from GP practices who were randomized in an intervention and a control group (Registration number: DRKS00012677). The control group was conducted as a waiting list control group, i.e., these participants received the intervention after the collection of follow-up data. The study aimed at evaluating the effectiveness of the IMPROVE*job* intervention on increasing job satisfaction as measured by the German version of the COPSOQ 2018 (primary outcome). The intervention lasted nine months, with baseline data collected immediately before the start of the intervention and follow‑up data collected approximately nine months later at the end of the intervention period.

The IMPROVE*job* trial was approved by the Ethics’ Committee of the Medical Faculty of the University of Bonn (reference number 057/19, date of approval: 20 February 2019). In addition, the Ethics Committees of the Medical Association North-Rhine (Lfd-Nr.: 2019107) and of the Medical Faculty, University Hospital of Tuebingen (Project-No.: 446/2019BO2) also voted positively. Each participant provided written informed consent.

### Recruitment and eligibility criteria

The study population of IMPROVE*job* consisted of practice personnel of general practices of the North Rhine Region in Germany. The eligibility criteria for practices included registration of their owner as a GP with the Association of Statutory Health Insurance Physicians of North Rhine, whether affiliated as a teaching practice of the University of Bonn or the University of Cologne or not. Additionally, the practice owner and at least one practice assistant required providing their informed consent for the study.

**Fig. 1:**
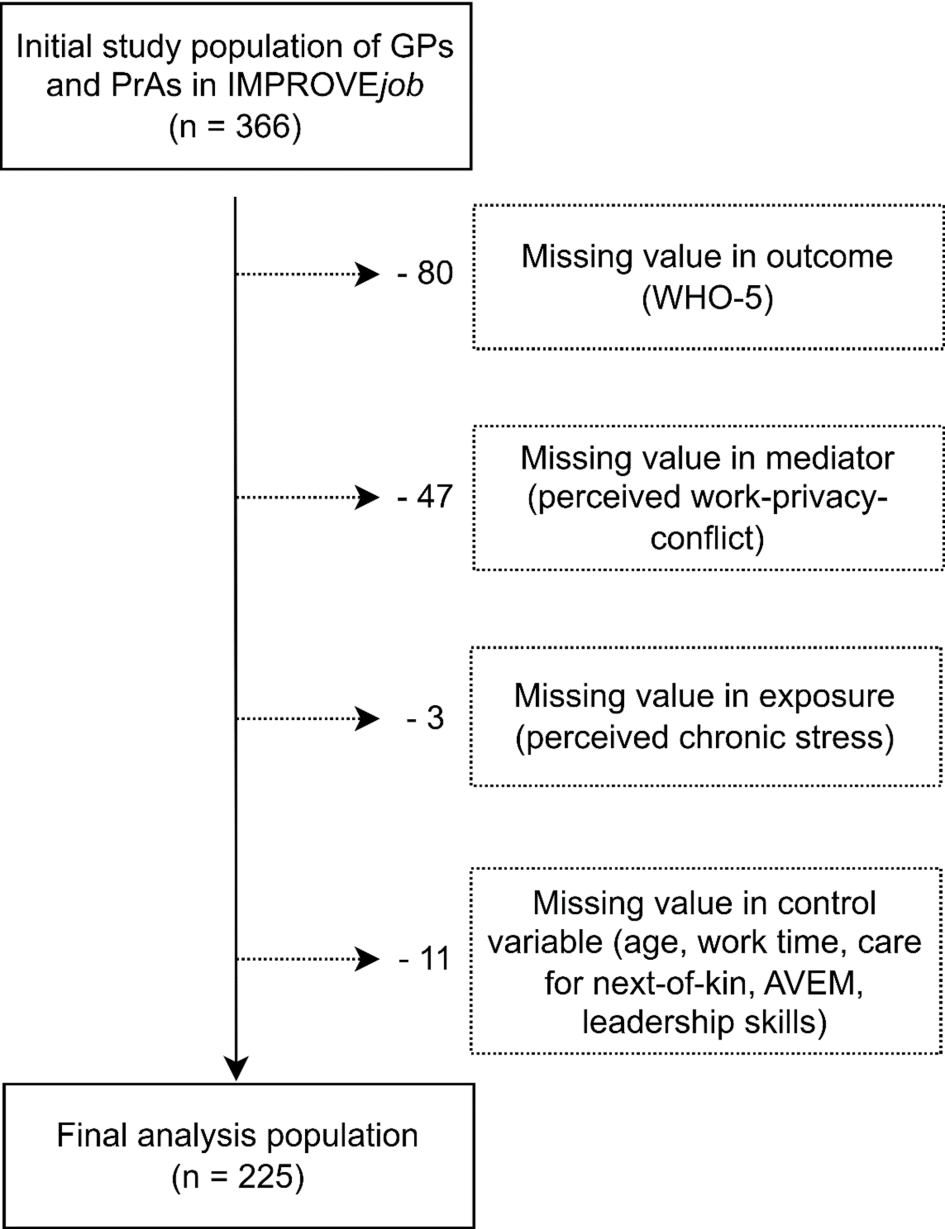
Analysis population based on the IMPROVE*job* study population of German general practice personnel

Randomization was conducted at practice level with practices acting as clusters. Sixty practices were recruited with 366 participants. With 84 (23%) physicians in a leadership position, 28 (7.6%) employed physicians, and 254 (69.3) practice assistants were included in the initial study population of the IMPROVE*job* data. We excluded participants with either missing values in any endogenous variable (work-privacy conflict, WHO-5), or in control variables when the number of missing values were low. The final analysis population covered 225 participants (Fig. 1). As missingness was low and compatible with a missing completely at random (MCAR) mechanism, we performed complete-case analyses and did not apply multiple imputations.

### Outcome variable: risk of incident depression during the study period

The primary outcome was the risk of developing new-onset depression between baseline and follow-up. To assess subjective well-being and screen for potential depressive symptoms, we used the 5-item World Health Organization Well-Being Index (WHO-5), a widely recognized and validated tool for evaluating mental health [20]. The WHO-5 is a concise global rating scale originally introduced at a WHO meeting in Stockholm in February 1998 as part of a project aimed at measuring well-being in primary health care patients. Since its introduction, the scale has been translated into multiple languages by the WHO Regional Office for Europe, enabling its widespread use in both clinical and research contexts. The WHO-5 consists of five positively phrased items that reflect various aspects of mental health and well-being, namely: feeling cheerful and in good spirits, feeling calm and relaxed, feeling active and vigorous, waking up feeling fresh and rested, and finding daily life filled with things that interest the respondent.

Respondents rate their experiences for the past 14 days on a scale ranging from 5 (all of the time) to 0 (none of the time), yielding a raw score between 0, indicating an absence of well-being, and 25, representing maximal well-being. To align with conventional health-related quality-of-life metrics, the raw score is typically converted to a percentage scale ranging from 0 to 100 by multiplying it by four. The tool has demonstrated strong validity as a screening instrument for depression, with a sensitivity of 0.86 and a specificity of 0.81. Threshold scores are used to identify individuals at risk; a score of ≤ 50 is indicative of poor well-being and serves as a “screening diagnosis” for depression, while a score of ≤ 28 is recommended when assessing the risk of depression onset [20–22].

In this study, individuals with pre-existing depression at baseline were excluded. Baseline depression was defined as WHO-5 ≤ 50 or a documented diagnosis. Among the remaining participants, incident depression was defined as WHO-5 ≤ 50 at follow-up. This threshold indicates poor well-being/possible depression and was used consistently for both baseline exclusion and follow-up incidence due to its established screening validity in primary care (sensitivity 0.86, specificity 0.81) [20–22]. A sensitivity analysis using the stricter ≤ 28 threshold yielded similar results.

### Mediator variable: the change in perceived work-privacy conflict during the study period

Perceived work-privacy conflict (WPC) is the mediator variable of interest and was assessed using the corresponding 2‑item scale from the 2018 German edition of the international Copenhagen Psychosocial Questionnaire (COPSOQ III version), a validated tool for gauging psychosocial aspects of the workplace. This scale consists of two statements (“The demands of my work interfere with my home and family life”; “The amount of time my job takes up makes it difficult to fulfill my family responsibilities”) and demonstrates strong reliability with Cronbach’s alpha coefficient of 0.92 [19]. Responses were categorized as strongly agree, somewhat agree, undecided, somewhat disagree, or strongly disagree. Following the COPSOQ manual, these responses were then converted into a numerical scale ranging from 0 to 100, with higher scores indicating greater levels of WPC. Change (Δ) was calculated as (*Δ = score*$\:{}_{follow-up}$ *− score*$\:{}_{baseline})$; positive values indicate worsening. For primary analyses, increases were dichotomized as Δ > 0 (increased) vs. Δ ≤ 0 (stable/decreased).

### Independent variable: change in perceived chronic stress during the study period

In this study, perceived chronic stress was assessed using the short version of the Trier inventory for chronic stress (TICS-SSCS, 12-item), a comprehensive instrument developed by Schulz et al. [23]. The TICS-SSCS is rooted in the systemic-requirement-resource model of health and is specifically designed to evaluate chronic stress with a high degree of content validity [24]. This tool is widely employed in Germany and provides a structured and reliable approach to measuring chronic stress across multiple domains. It comprises 57 items that assess nine specific domains related to chronic stress, including work overload, social isolation, pressure to perform, social overload, excessive work demands, work discontent, lack of social recognition, social tensions, and chronic worrying. Additionally, a shorter screening scale, known as the Short Screening Scale for Chronic Stress (TICS-SSCS), was derived from a representative sample of the German population (*N* = 604) [25]. This version consists of 12 items selected from five of the nine stress domains: chronic worrying, work overload, social overload, excessive work demands, and lack of social recognition. Respondents used a 5-point Likert scale to indicate the frequency of their experiences for each stress-related item, ranging from 0 (“never”) to 4 (“very often”). The TICS-SSCS assesses perceived chronic stress experienced over the past three months, enabling a focus on recent stressors. It is a standardized and validated tool, demonstrating strong internal consistency with a Cronbach’s Alpha coefficient of 0.91 for the 12-item TICS-SSCS, indicating high reliability. Individual item reliabilities range from 0.84 to 0.91, with a mean alpha of 0.87, reinforcing the instrument’s robustness and consistency [23, 24]. In the DEGS1 study from Robert Koch institute [14], TICS-SSCS scores were categorized as low stress (≤ 11), medium stress (12–22), and high stress (> 22, 90th percentile). However, our GP personnel showed elevated stress levels with a sample median of 19 (vs. population median 11). TICS-SSCS scores were therefore dichotomized at the sample median (≤ 19 vs. > 19) to better reflect this occupational group’s distribution (Figure S1 shows the distribution). This data-driven approach was preferred over population norms given the specific stress profile of general practice personnel. Sensitivity analyses using DEGS-1 cut-offs (> 22) yielded consistent results. Change (Δ) was calculated as (*Δ = score*$\:{}_{follow-up}$ *− score*$\:{}_{baseline})$; positive values indicate worsening. For primary analyses, increases were dichotomized as Δ > 0 (increased) vs. Δ ≤ 0 (stable/decreased).

; positive values indicate worsening. For primary analyses, increases were dichotomized as Δ > 0 (increased) vs. Δ ≤ 0 (stable/decreased).

### Control variables and further characteristics of interest

As potential confounding personal and work-related factors such as age, gender, work time (full-time/part-time), marital status, care for next-of-kin (yes/no), and leadership skills (LMX-7) were included in the mediation analysis (Table 1). Additionally, we analyzed in a descriptive step the dimensions of work-related behaviour (AVEM) using the short version of 44-item AVEM identifies eleven work-related behavioural and experience dimensions from the following three area [26, 27]:

- *engagement with work* (subjective importance of work, work-related ambition, willingness to work until exhausted, striving for perfection, and distancing ability),
- *resilience* in dealing with the everyday stress of work (distancing ability, tendency to resignation in the face of failure, proactive problem-solving, and inner calm and balance), and.
- *emotions* associated with work and life in general (experience of success at work, satisfaction with life, and experience of social support).

Distancing ability is an important component of both the first (negative correlated for engagement) and second area (positive correlated for resilience) [26, 27].

### Statistical analysis

Participants were clustered within 60 practices randomized to intervention vs. waiting-list control. As reported in the main IMPROVEjob paper [18], the intervention showed no significant effects on job satisfaction, stress, or well-being outcomes [18]. Trial arm was tested in all models and showed no main effects or interactions; results are presented pooled across arms. Within-person change scores (Δ) implicitly adjust for time-invariant practice effects. All regression and mediation models used cluster-robust standard errors at practice level.

In a first step, the mental burden of general practice personnel was analysed in a descriptive way by looking at the status (baseline & follow-up) and change in perceived chronic stress (TICS-SSCS), risk of incident depression (WHO-5), work-privacy conflict (COPSOQ-WPC), job satisfaction (COPSOQ-JS) between baseline and follow-up.

In a second step, a single path analysis was conducted, in which the assumed pathway from perceived chronic stress over WPC on risk of incident depression were decomposed into the first path between stress and WPC and the second path between WPC on risk of incident depression.

In a third step, a causal mediation analysis was conducted to explore within the same model the interrelationships between perceived chronic stress change, work-privacy conflict change, and incident depression, assuming the causal chain: stress change → WPC change → depression. Causal mediation analysis is a newer approach to mediation analysis developed in the last decade, simulating an experimental design by applying a counterfactual framework [28–30]. Causal effects are defined as the disparity between two counterfactual outcomes. A counterfactual outcome refers to the hypothetical outcome value of an individual under a specific exposure condition (treatment vs. no treatment) [28–30].

We adopted the annotation from Imai et al. [29]. $\:{M}_{i}\left(t\right)$ denotes the potential value of a mediator for a unit $\:i$ under the treatment status $\:{T}_{i}=\:t$ (possible treatment status $\:\mathrm{t}=\mathrm{0,1}$). $\:{Y}_{i}(t,\:m)$ denotes the potential outcome resulting if the treatment and mediating variables are equal $\:t$ and $\:m$. Let us now imagine an experimental design, in which the treatment variable was randomized and we observe only one of the potential outcomes. The observed outcome, $\:{Y}_{i}$, can be written as $\:{Y}_{i}({T}_{i},\:{M}_{i}\left({T}_{i}\right))$ where $\:{M}_{i}\left({T}_{i}\right)$ is the observed value of the mediator $\:{M}_{i}$. Accordingly, the total effect, is

1 $$\:{{\uptau\:}}_{i}\equiv\:\:{Y}_{i}\left(1,\:{M}_{i}\left(1\right)\right)-{Y}_{i}(0,\:{M}_{i}(0\left)\right)$$

This total effect can be then decomposed into the average causal mediation effects (ACME) and the average direct effects (ADE). The ACME can be expressed as

2 $$\:{\delta\:}_{i}\left(t\right)\equiv\:{Y}_{i}\left(t,\:{M}_{i}\left(1\right)\right)-{Y}_{i}(t,\:{M}_{i}(0\left)\right)$$

All other (unobserved) causal mechanisms, either direct or indirect ones, are summarized in the average direct effects (ADE) of the treatment

3 $$\:{\zeta\:}_{i}\left(t\right)\equiv\:{Y}_{i}\left(1,\:{M}_{i}\left(t\right)\right)-{Y}_{i}(0,\:{M}_{i}(t\left)\right)$$

Control variables were denoted with $\:C$. To apply causal mediation analysis, we used the mediation package in R [31]. The analyses were conducted on R 4.1.1 [32]. Specifically, the mediator model was specified as LM (Δ WPC ~ increased stress + covariates) and the outcome model as GLM (incident depression ~ increased stress + Δ WPC + covariates, family = binomial). Covariates included age, gender, professional role, full-time status, marital status, care for next-of-kin, LMX-7 leadership score, trial arm, and baseline values of TICS-SSCS, WPC, and WHO-5. The analysis assumes sequential ignorability (no unmeasured confounding conditional on covariates). An exposure-mediator interaction was tested and found non-significant. The counterfactual framework decomposes the total effect into average causal mediation effect (ACME) and average direct effect (ADE) [28–30]. The analyses were conducted on R 4.1.1 [32].

## Results

### Descriptive results

The final sample of 225 participants were mostly females (*n* = 195, 86.7%), and 13.3% were males. The mean age of the total sample was 44.3 ± 12.3 years. 69.8% were general practice assistants, with more than half of the participants (54.7%) reported to work full-time. most of the participants were living in a relationship or married (80.8), and only 21% answered yes to care for next of kin. Further details on these subgroups are shown in Table 1.

**Table 1:** Sociodemographic characteristics of the general practice personnel

Variable	Total sample (*n* = 225)
Female, n (%)	195 (86.7)
Age in years, mean [SD]	44.3 [12.3]
Physicians, n (%)	68 (30.2)
General practice assistants, n (%)	157 (69.8)
Working full-time, n (%)	123 (54.7)
Living in a relationship/married, n (%)	181 (80.8)
Working years, mean [SD]	19.6 [13.1]
Care for next-of-kin, n (%)	47 (21)
Control group, n (%)	118 (52.4)
Leadership, mean [SD]	26.9 [4.1]

Table 2 displays the descriptive results of various variables at baseline and follow-up, along with the differences between baseline and follow-up (Δ) assessments. The average scores for perceived chronic stress and well-being significantly decreased, while WPC scores increased.

**Table 2:** Descriptive results of chronic stress (TICS-SSCS), risk of incident depression (WHO-5), Work-Privacy conflict (COPSOQ-WPC), job satisfaction (COPSOQ-JS): baseline, follow-up, and differences between baseline and follow-up (Δ)

Variable	Mean [SD] or *n* (%)			t-test (*p*-value)
	Baseline *N* = 225	Follow-up *N* = 225	Delta*	
TICS*	19.00 [8.00]	17.94 [8.4.0]	-1.10 [7.00]	2.30 (0.023)
Below average stress (0–19)	116 (51.6)	136 (60.4)		
Above average stress (20–48)	109 (48.4)	89 (39.6)		
WHO-5*	55.59 [21.50]	51.77 [22.70]	-4.05 [19.15]	3.15 (0.002)
Good well-being (51–100)	146 (64.9)	124 (55.1)		
Poor well-being (≤ 50)	76 (33.8)	100 (44.4)		
Not reported	3 (1.3)	1 (0.4)		
COPSOQ-WPC	41.06 [31.53]	46 [30.54]	4.94 [25.58]	-2.90 (0.004)
COPSOQ-JS	74.41 [13.22]	72.81 [15.78]	-1.60 [12.85]	1.87 (0.063)

*Δ = follow‑up value - baseline value; positive values indicate an increase over time; Below average stress: TICS‑SSCS score 0–19; above average stress: 20–48; Good well‑being: WHO‑5 score 51–100; poor well‑being: ≤50

Among the participants, 43.64% experienced increased levels of perceived chronic stress from baseline to follow-up, while 56.36% reported stable or decreased stress levels. Additionally, 43.22% of individuals demonstrated increased work-privacy conflict, with 28.39% maintaining stable work-privacy conflict levels and another 28.39% reporting a decrease. Regarding incident depression (WHO-5 ≤ 28), 19.49% of participants developed depression during the study period, whereas 80.51% did not experience any depressive symptoms.

Table 3 provides a summary of the expressions of the individual AVEM dimensions (interval scale Stanine scores). Participants reported the highest mean scores in experience of social support (M=16.3 (3.4)) and satisfaction with life (M= 16.0 (2.8)), indicating generally high levels of life satisfaction and perceived social support. In contrast, the lowest mean scores were observed in subjective importance of work (M=9.3 (3.6)) and tendency to resign in the face of failure (M=9.5 (3.3)), suggesting these areas may be less emphasized or more challenging for participants. Other notable dimensions include work-related ambition (M=11.7 (3.7)) and willingness to work until exhausted (M=12.0 (3.9)), reflecting moderate levels of ambition and work intensity. Overall, participants exhibited positive coping strategies and resilience, as reflected in higher mean scores for striving for perfection (M=13.6 (3.5)), distancing ability (M=13.6 (3.9)), proactive problem-solving (M=13.0 (3.0)), and inner calm and balance (M=13.3 (3.3)). This aligns with findings from our previous study, which identified a positive selection bias among general practitioners, demonstrating their ability to develop resilience and effective coping mechanisms. These results highlight their capacity to mitigate the negative effects of chronic stress despite commonly emphasized adverse outcomes.

**Table 3:** Work characteristics of the AVEM dimensions

AVEM Dimension	Mean [SD] (*n* = 225)
Subjective importance of work	9.3 [3.6]
Work-related ambition	11.7 [3.7]
Willingness to work until exhausted	12.0 [3.9]
Striving for perfection	13.6 [3.5]
Distancing ability	13.6 [3.9]
Tendency to resign in the face of failure	9.5 [3.3]
Proactive problem-solving	13.0 [3.0]
Inner calm and balance	13.3 [3.3]
Experience of success at work	14.2 [3.3]
Satisfaction with life	16.0 [2.8]
Experience of social support	16.3 [3.4]

### Single path analysis: the interrelationships between perceived chronic stress, work-privacy conflict, and risk of incident depression among GP practice personnel

The linear regression model (Table 4) for work-privacy conflict revealed that GP personnel, who increased perceived chronic stress, reported stronger increase in work-privacy conflict than those who perceived no change or a decrease in chronic stress (β = 8.99, *p* = 0.01). Participants with higher baseline levels of work-privacy conflict were less prone to increases in work-privacy conflict (β = -0.35, *p* < 0.001). All eleven AVEM dimensions showed no significant correlation with the change in work-privacy conflict.

In the logistic regression model for risk of incident depression, increased work-privacy conflict was associated with a higher risk of incident depression (logit β = 0.20, *p* = 0.015; OR = 1.02 per point, 1.22 per 10-point increase). Participants with higher baseline levels of work-privacy conflict were less prone to increases in work-privacy conflict (β = -0.35, *p* < 0.001). All eleven AVEM dimensions showed no significant association with the risk of incident depression.

**Table 4:** Results of the mediation analysis with decomposition of the total effect into the direct and indirect effects

Model *	Outcome	Predictor	Coefficient (β)	OR	*p*-value
Single-path, linear	Δ WPC	Increased perceived chronic stress (ref. no increase/decrease)	8.986		0.004
Single-path, logistic	Incident depression	Δ WPC (per 1-point increase)	0.020	1.02 per 1-point; 1.22 per 10-point increase	0.015
Mediation – total effect	Incident depression	Increased perceived chronic stress	(logit) 0.194	exp(0.194) ≈ 1.21	0.002
Mediation – direct effect (ADE)	-	-	0.168	≈ 1.18	0.002
Mediation – indirect effect (ACME)	-	-	0.026	≈ 1.03	0.020

*Linear model (LM) used for ΔWPC; logistic regression (LR) used for incident depression

### Causal mediation analysis: the role of work-privacy conflict in the association of perceived chronic stress and the risk of incident depression

The total effect of the mediation analysis (Table 4) revealed a significant association between perceived chronic stress and risk of incident depression: If perceived chronic stress has increased during the study period among GPs or PrAs the risk of incident depression was higher compared to participants who perceived no increase or a decrease in stress (Total Effect: $\:{{\uptau\:}}_{i}$ = 0.19, *p* = 0.002).

**Fig. 2:**
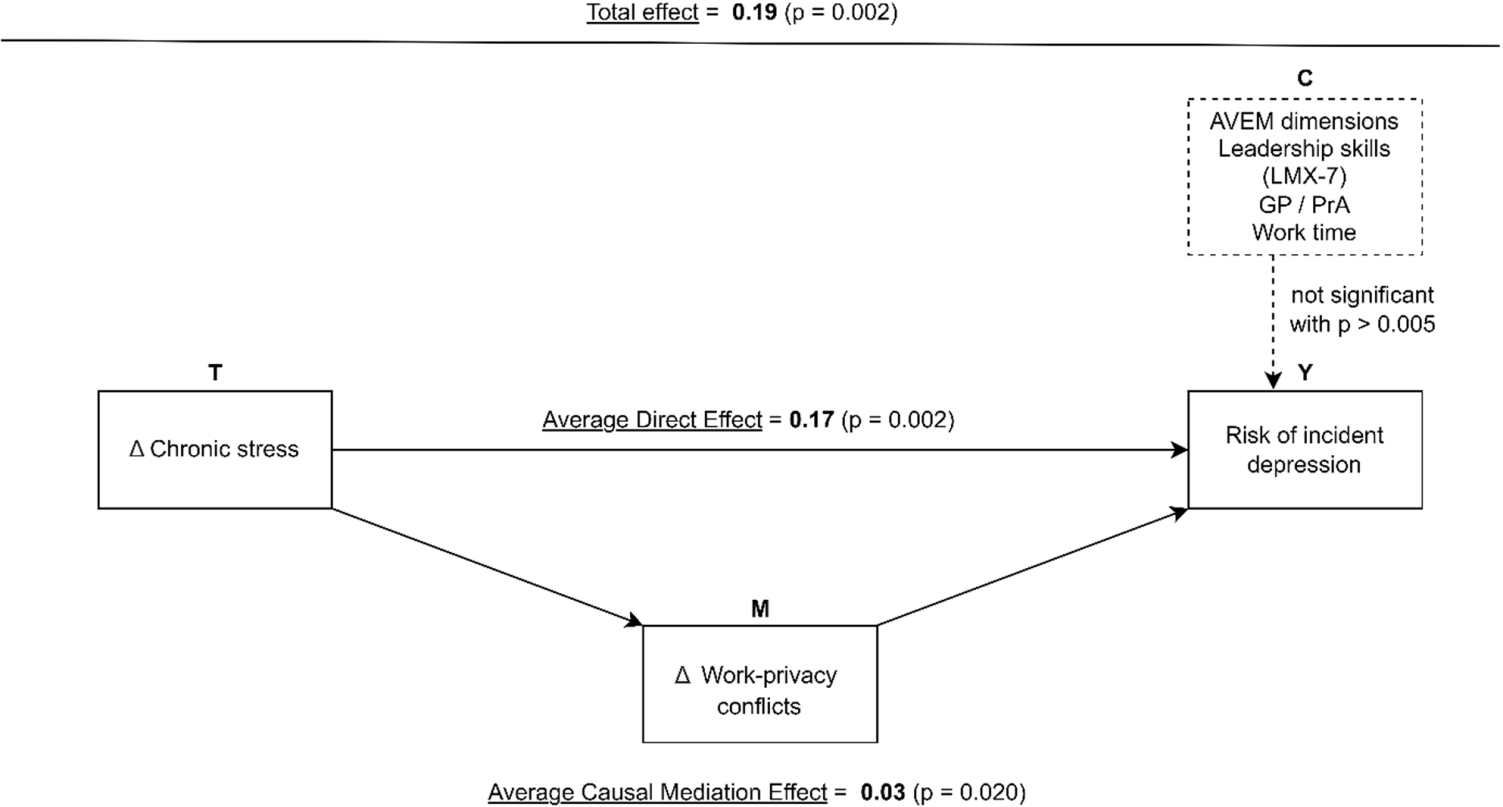
The estimated causal mediation model and its results

When we decomposed the total effect both a significant average direct effect ($\:{\zeta\:}_{i}$ = 0.17, *p* = 0.002) and a causal mediation effect ($\:{\delta\:}_{i}$= 0.03, *p* = 0.020) were observed (Fig. 2). This suggested partial mediation and that the increased risk of incident depression associated by perceived chronic stress was in part translated by a higher degree of work-privacy conflict in GP practice personnel.

We did not find any significant association between personal or other work-related factors such as age, gender, work time, marital status, AVEM dimensions, care for next-of-kin, LMX-7, and the risk of incident depression.

## Discussion

### Key findings

This study investigated the associations between the risk of incident depression, chronic stress, and work-privacy conflict among German general practice personnel using a causal pathway approach. Increased chronic stress was associated with a higher risk of incident depression supporting our hypothesis that stress is a crucial factor influencing mental health outcomes in GPs and PrAs. This finding aligns with existing literature emphasizing the detrimental impact of chronic stress on mental health [33, 34].

When decomposing the total effect, we found a significant average direct effect of chronic stress on the risk of incident depression, and a causal mediation effect via the pathway over work-privacy conflict. The direct effect suggests that chronic stress directly contributes to depression, independent of other controlled factors in the model. The causal mediation effect indicates that work-privacy conflict partially mediates the relationship between chronic stress and risk of incident depression. While previous literature reported that the increased work-privacy conflict can be explained by several factors such as higher workload, management issues, and ongoing regular patient care services [35]. This partial mediation suggests that while chronic stress directly affects depression, some of its impact is translated through increased work-privacy conflict. This finding is of great importance and contributes to understanding the association between work-privacy conflict and risk of incident depression in primary care setting (Zhang et al., 2023). Furthermore, the partial mediation through work-privacy conflict is particularly relevant for interventions; it suggests that addressing work-privacy conflict could mitigate some of the adverse effects of chronic stress on risk of incident depression. Work-privacy conflict may exacerbate the perception of stress by limiting the ability of GP personnel to disengage from work-related pressures during personal time, thereby increasing their overall stress load and risk of incident depression [36].The IMPROVE*job* study was conducted during the COVID-19 pandemic, a period marked by heightened stress and significant changes in work dynamics, particularly in healthcare settings. The pandemic likely intensified the perceived chronic stress among GP personnel, as they faced increased workload, higher risk of infection, and greater uncertainty [37]. These extraordinary conditions could have amplified the effects of chronic stress and work-privacy conflict on risk of incident depression, suggesting that our findings might be particularly relevant in the context of crises.

Unlike what has been reported by Göbel et al. [16], where the work-privacy conflict differed between practice owners and employer physicians, our results showed no significant effect of personal factors in the association between chronic stress and risk of incident depression when mediated by work privacy conflict. Additionally, AVEM dimensions of work-related behavior and experience patterns did not show significant effects in our analysis, likely because they do not capture the specific stressors relevant to GP personnel during the COVID-19 pandemic. Unique challenges introduced by the pandemic may not be reflected in traditional patterns [38]. In our previous research, we revealed a predominance of health-promoting AVEM patterns among general practice teams but also emphasized the need for structural prevention and behavioral health promotion to address work-related stress [6]. Additionally, the chronic stress and work-privacy conflict during this period could have overshadowed other behavior patterns. The AVEM instrument, developed in a different era, may be outdated and fail to encompass contemporary occupational stressors faced by healthcare professionals under pandemic conditions. Future research should develop or adapt tools that are more context-specific to accurately assess stressors and their impact on GP personnel’s well-being.

### Limitations and strengths

The data analysed in this study was collected from the complete general practice team rather than one subgroup, which strengths the methodology and supports generalizing the results obtained. In this study, several limitations should be acknowledged. A notable limitation is the use of the WHO-5 Well-Being Index as the primary measure for assessing risk of incident depression. While the WHO-5 is a subjective screening tool for overall well-being, its application mainly pertains to depression risk assessment in general practice (GP) settings, thus its inclusion in our study. However, the WHO-5’s focus on well-being may underestimate risk of incident depression prevalence, especially in individuals with mild or atypical symptoms that do not significantly affect their well-being. It also does not distinguish between different subtypes of risk of incident depression, which limits insights into how chronic stress and work-life conflict affect various depressive disorders. The assumed direction from chronic stress to WPC is theoretically plausible but cannot exclude reverse or reciprocal causation, so the findings should be interpreted as associations along one possible pathway rather than proof of a strictly unidirectional causal chain. Leadership quality (LMX‑7) and working time (full‑ vs. part‑time) showed no significant association with incident depression, which contrasts with some prior studies in healthcare settings; this discrepancy may reflect limited statistical power due to the relatively small number of incident cases, generally high leadership quality in the participating practices, and the predominance of pandemic‑related stressors during the study period. Moreover, important potential confounders such as education level, disease history, and lifestyle factors (e.g. smoking, alcohol use, physical activity) were not available in our data and should be considered in future studies.

The longitudinal two-wave design of the study allows examination of temporal associations between perceived chronic stress, work‑privacy conflict, and risk of incident depression, but still limits strong causal inference because only two measurement points and observational data are available. Longitudinal studies are needed to better understand these temporal dynamics and causal relationships. Additionally, the unique stressors introduced by the COVID-19 pandemic may have influenced our findings’ generalizability.

Our study highlights the need to address unmeasured confounding in future research. Ensuring transparency by detailing empirical approaches or sensitivity analyses used to validate identifiability assumptions is crucial. Accurate measurement and validation of mediators are essential to mitigate potential biases. Discussions should also consider non-causal explanations, such as lack of statistical power or insufficient sample size, and report on their potential influence.

We recommend incorporating exposure-mediator interactions in future studies. Presenting results from models with and without interaction terms can provide a comprehensive understanding of the relationships and potential moderating effects between exposure and mediator. By addressing these recommendations, future research can enhance the robustness and credibility of findings in studies investigating mediator-outcome relationships.

## Conclusion

This study highlights the role of work-privacy conflict in the mental health burden of general practice personnel due to perceived chronic stress. Future interventions should address the development of work-privacy conflict to overcome the high burden of chronic stress among general practitioners and practice assistants.

## Data Availability

There are no plans to grant access to full protocol, participant-level datasets, or statistical codes, as data contain potentially identifying information.

## Abbreviations

TICS-SSCS: Chronic Stress Screening Scale
COPSOQ III: Copenhagen Psychosocial Questionnaire
cRCT: Cluster-randomized controlled trial
GPs: General practitioners
PrAs: Practice assistants
WPC: Work-privacy conflict
WHO-5: World Health Organization Well-Being Index
AVEM: Work-related behaviour
